# Understanding work-related travel and its relation to malaria occurrence in Thailand using geospatial maximum entropy modelling

**DOI:** 10.1186/s12936-023-04478-6

**Published:** 2023-02-13

**Authors:** Natalie Memarsadeghi, Kathleen Stewart, Yao Li, Siriporn Sornsakrin, Nichaphat Uthaimongkol, Worachet Kuntawunginn, Kingkan Pidtana, Chatree Raseebut, Mariusz Wojnarski, Krisada Jongsakul, Danai Jearakul, Norman Waters, Michele Spring, Shannon Takala-Harrison

**Affiliations:** 1 Department of Geographical Sciences, University of Maryland, College Park, MD 20742 USA; 2 Armed Forces Research Institute of Medical Sciences (AFRIMS), Bangkok, Thailand; 3 Division of Vector Borne Diseases, Ministry of Public Health, Ubon Ratchathani, Thailand; 4 Center for Vaccine Development and Global Health, University of Maryland School of Medicine, Baltimore, MD USA

## Abstract

**Background:**

Estimating malaria risk associated with work locations and travel across a region provides local health officials with information useful to mitigate possible transmission paths of malaria as well as understand the risk of exposure for local populations. This study investigates malaria exposure risk by analysing the spatial pattern of malaria cases (primarily *Plasmodium vivax)* in Ubon Ratchathani and Sisaket provinces of Thailand, using an ecological niche model and machine learning to estimate the species distribution of *P. vivax* malaria and compare the resulting niche areas with occupation type, work locations, and work-related travel routes.

**Methods:**

A maximum entropy model was trained to estimate the distribution of *P. vivax* malaria for a period between January 2019 and April 2020, capturing estimated malaria occurrence for these provinces. A random simulation workflow was developed to make region-based case data usable for the machine learning approach. This workflow was used to generate a probability surface for the ecological niche regions. The resulting niche regions were analysed by occupation type, home and work locations, and work-related travel routes to determine the relationship between these variables and malaria occurrence. A one-way analysis of variance (ANOVA) test was used to understand the relationship between predicted malaria occurrence and occupation type.

**Results:**

The MaxEnt (full name) model indicated a higher occurrence of *P. vivax* malaria in forested areas especially along the Thailand–Cambodia border. The ANOVA results showed a statistically significant difference between average malaria risk values predicted from the ecological niche model for rubber plantation workers and farmers, the two main occupation groups in the study. The rubber plantation workers were found to be at higher risk of exposure to malaria than farmers in Ubon Ratchathani and Sisaket provinces of Thailand.

**Conclusion:**

The results from this study point to occupation-related factors such as work location and the routes travelled to work, being risk factors in malaria occurrence and possible contributors to transmission among local populations.

## Background

Malaria has been a public health priority of the Greater Mekong Subregion for years, although in recent years, high treatment failure rates in *Plasmodium falciparum* in eastern Thailand have necessitated rapid policy responses. To address the concerning rates of antimalarial drug resistance, In 2020, Thailand’s Ministry of Public Health changed the first-line therapy for *P. falciparum* to artesunate-pyronaridine (AS-PY) in Sisaket, to address high treatment failure rates [1]. This resulted in a steep decline of *P. falciparum* cases to the point where *Plasmodium vivax* has become the predominant species causing malaria cases in Thailand, with a much smaller proportion of *P. falciparum* cases. Many of these cases have been clustered along international borders, including the borders with Myanmar and Cambodia [2]. However overall, malaria prevalence has been decreasing in recent years, and Thailand has a goal to eliminate malaria by 2024. From 2012 to 2017, there was a 67% decline in the number of cases including an accelerated decline of 39% from 2016 to 2017 [3]. While Thailand is moving forward with its elimination goals, there are still significant pockets of malaria in Thailand as recent reports [4] indicate that malaria cases have doubled in fiscal year 2022 compared with the previous fiscal year.

Estimating malaria risk across a region provides local health officials with information useful for the mitigation of possible local transmission paths, and for understanding the risk of exposure for local populations. In this study, a machine learning approach was utilized to estimate the spatial distribution of malaria in two provinces in eastern Thailand, Ubon Ratchathani and Sisaket, where both *P. vivax* and *P. falciparum* had been found in clinical studies and was being monitored by a team from the United States Armed Forces Research Institute of Medical Sciences (AFRIMS). The research objective was to estimate the association of occupation-related factors, including occupation type and work locations, with estimated malaria occurrence in these two provinces to understand how local populations may be vulnerable to malaria based on where they reside, where their workplace is located, and the routes they take to reach their workplace. Malaria occurrence here refers to the probability of having malaria at a location.

To generate an estimate of the spatial variability of malaria occurrence within these provinces, a maximum entropy-based modelling tool (MaxEnt) was used to model the malaria species distribution. MaxEnt is an ecological niche modelling tool that predicts species distribution across a defined geographic area using case data and relevant environmental variables [5]. The true distribution of a species is represented as a probability distribution that considers the constraints of incidence data in relation to the empirical average of environmental data at sites where cases are detected [6–8]. MaxEnt produces a probability map showing whether a species existed in a location or not, and this tool was used to predict the species distribution of *P. vivax* malaria for the period, January 2019 to April 2020 in Ubon Ratchathani and Sisaket. Environmental and sociodemographic variables were used to train the ecological niche model, including precipitation, temperature, elevation, land cover and population density as well as the locations of known local positive *P. vivax* and *P. falciparum* cases in these two provinces. These variables were used to determine the suitable environmental conditions for local malaria transmission and to create a probability surface that captures the ecological niche, reflecting the likelihood of malaria occurrence across the study area. The relationship between the probability of malaria occurrence and occupation type, work-related locations, as well as estimated travel routes between home and work locations (at village-level granularity) has been analysed to better understand the impact that residential and work locations, as well as work-related travel have on risk of exposure in outdoor settings and local transmission of malaria within this study area.

Several studies have used an ecological niche modelling approach to model the species distribution of *Anopheles* mosquitoes. Studies where the maximum entropy tool was used to determine the spatial distribution of the local *Anopheles* mosquito species have been undertaken in regions all over the world, including Asia and the Middle East [9], South America [10, 11], and Africa [12, 13]. MaxEnt has also been used to model the effects of climate change on malaria transmission and *Anopheles* species density in China [14], Iran [15], and parts of Africa [16]. In the model, local *P. vivax* malaria cases were used to train the machine learning model and estimate the percent probability of malaria occurrence given certain geographical and population factors.

The correlation between demographic variables and malaria risk has been previously studied, for example, an investigation of malaria risk factors in northern Namibia used survey data of malaria positive and negative individuals to identify significant risk factors for malaria in Namibia. In this study, cattle herders and police were the occupation types at the highest risk, and cross-border travel, open sleeping quarters, and working in agriculture overnight had a significant relationship with malaria infection [17, 18]. Significant risk factors for *P. vivax* malaria infections in Western Thailand included previous history of clinical malaria, occupation in agriculture, and travel to Myanmar [19]. In further studies, malaria prevalence has been very high in forest and forest fringes along rural stretches of border areas in Thailand, and forest workers, and local migrants were the individuals at highest risk [20–23]. Researchers found that challenges for malaria control existed especially in border regions with highly mobile migrant workers [24]. Recent research findings showed that limited vector protection for forest goers was one of the main reasons underlying malaria transmission hotspots located in the border areas [25, 26]. As occupation and work-related travel were expected to play a key role in exposure to malaria infection [25], this study focused on how different environmental factors impact the spatial distribution and extent of the ecological niche in Ubon Ratchathani and Sisaket, a *P. vivax* transmission prone area of eastern Thailand.

## Methods

### Data and study area

**Fig. 1:**
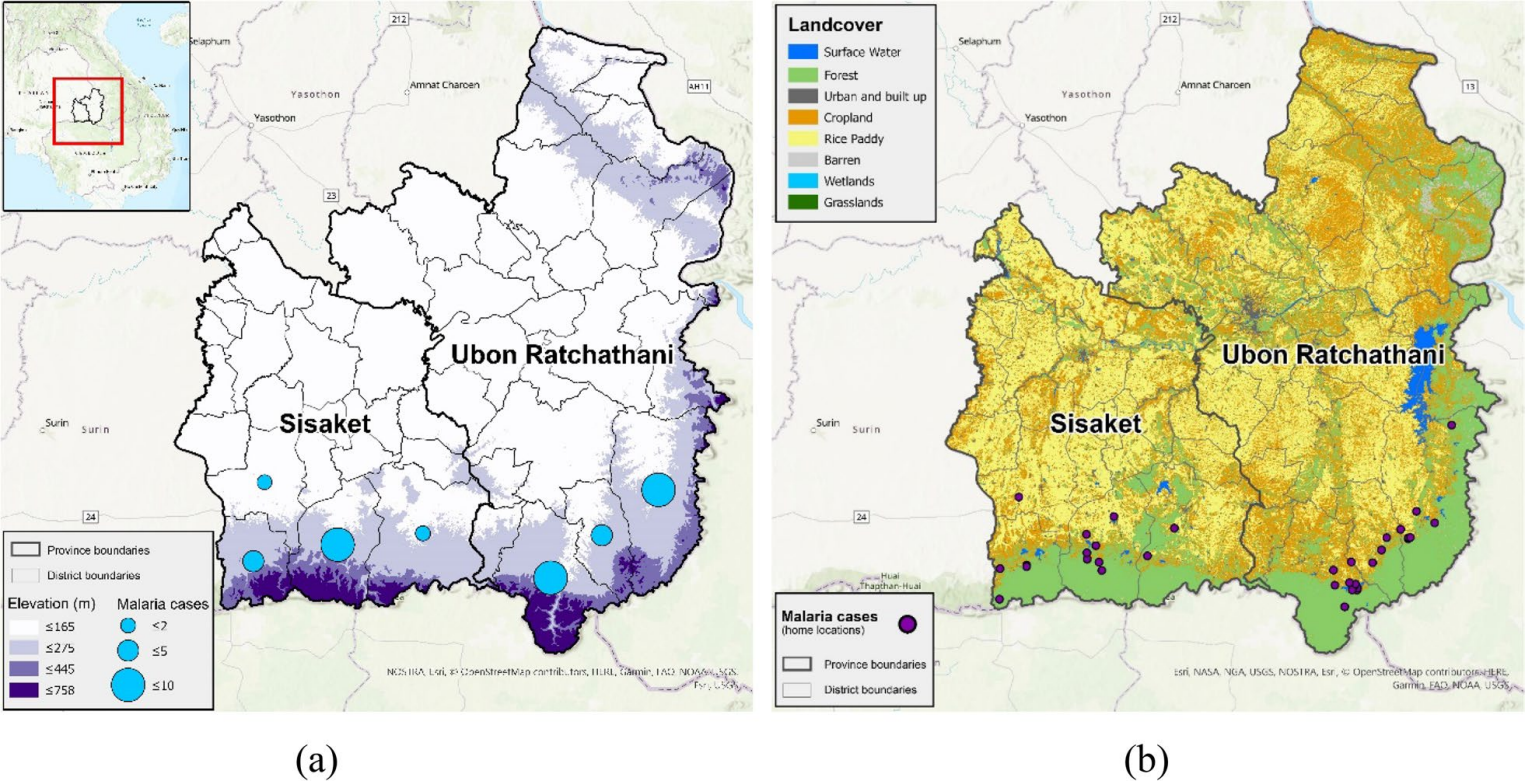
**a** Study area of Ubon Ratchathani and Sisaket, Thailand and the malaria cases per district in study sample enrolled by AFRIMS, and **b** landcover from SERVIR KEKONG Land Cover Portal and home village locations for malaria cases from data provided by AFRIMS

Ubon Ratchathani and Sisaket were selected based on an ongoing collaboration with researchers at the United States Armed Forces Research Institute of Medical Sciences (AFRIMS) based in Bangkok, Thailand who were conducting clinical studies of malaria in these two provinces (Fig. 1a).

Travel histories and other demographic data were collected by the AFRIMS research team as part of an ongoing study from March 2019 to April 2020 from 40 individuals reporting to malaria clinics and posts in Ubon Ratchathani and Sisaket. Polymerase chain reaction tests were applied to all samples to confirm speciation. Nearly all cases (39 cases) were diagnosed as *P. vivax.* (Fig. 1a). Due to the single case of *P. falciparum* that was reported, the study sample data is referred collectively as "malaria case data,” with the majority of cases being *P. vivax*. The survey participants met several requirements to be selected as participants in the study by AFRIMS, namely they were males or non-pregnant females, were older than 18 years and were civilian or military. They understood spoken Thai or a local language. In the survey, participants were asked to name their (permanent) home village to confirm that the data represent a local population. Other data used in this study included variables such as malaria diagnosis, age, sex, occupation type, and GPS coordinates for work locations and travel distance to work. For the AFRIMS survey data, the GPS coordinates were obtained for an approximate location near to their permanent residence in the village where they reside, and for work locations the approximate geographic coordinates capturing a close location were obtained for where they worked. This information was used in the analysis of occupation and work-related travel in relation to the estimated probability of malaria occurrence.

A landcover product for Myanmar, SERVIR KEKONG Land Cover Portal, was used for ecological niche modelling [27]. Based on the dataset, the primary land cover classes for Ubon Ratchathani and Sisaket provinces were forested areas, crop lands, and rice paddy fields. There is an escarpment with higher elevation (peak of 761 m) that is highly forested along the border of both provinces with Cambodia (Fig. 1b). This escarpment area has very low population density, however many of the surveyed individuals regularly travelled into this region for their work.

**Fig. 2:**
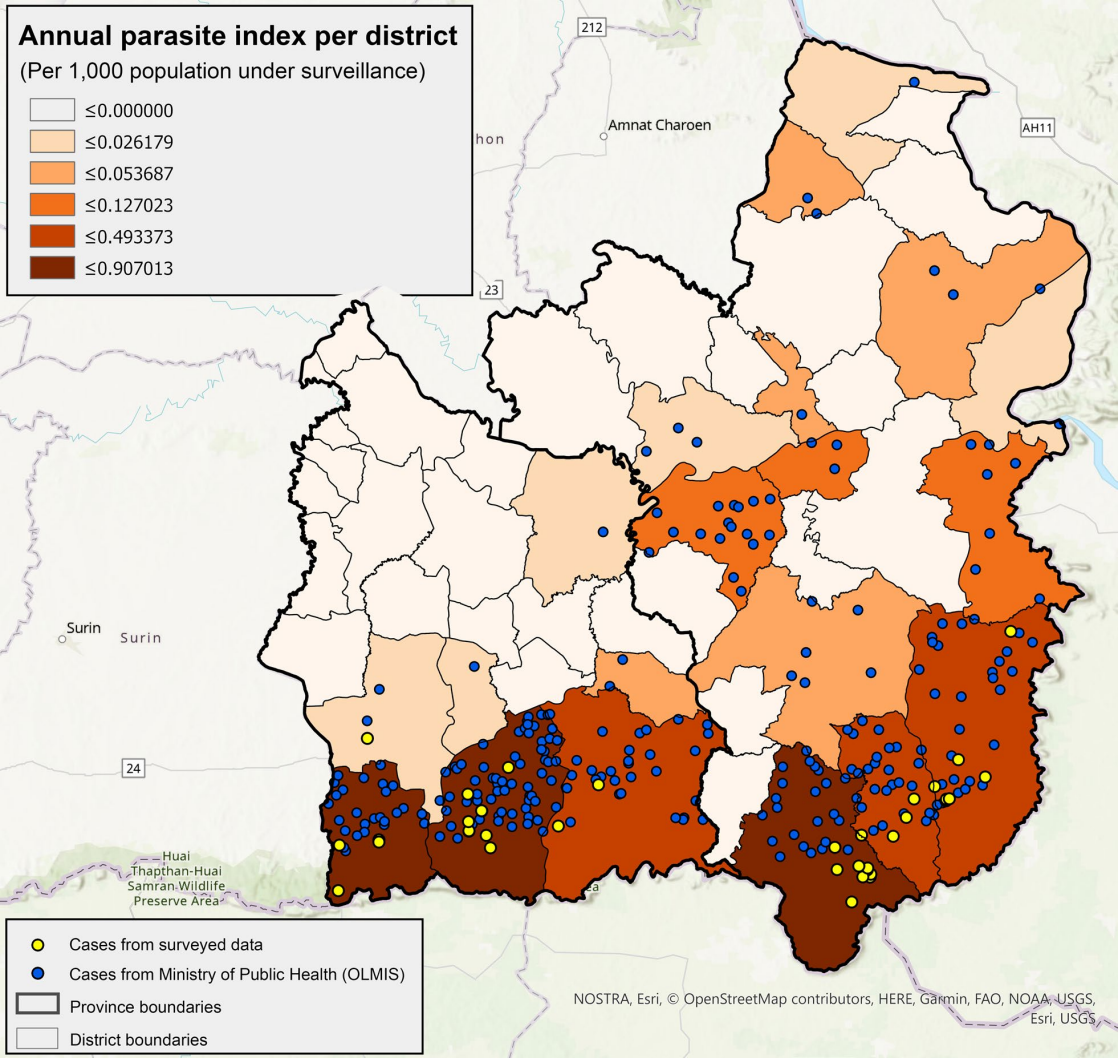
Annual parasite index by district recorded by the OLMIS between January 2019 and April 2020 classified using natural breaks overlaid with both simulated home village locations from OLMIS, and recorded home village locations from AFRIMS survey

In addition to the research data collected by AFRIMS, the malaria case data from the online malaria information system (OLMIS) managed by the Ministry of Public Health for Thailand (MOPH) was also obtained (Fig. 2). These data included the number of *P. vivax* and *P. falciparum* malaria cases in each district for Ubon Ratchathani and Sisaket from January 2019 to April 2020. The MOPH recorded 125 *P. vivax* malaria cases in Ubon Ratchathani and 201 cases in Sisaket, for a total of 326 *P. vivax* cases within the two provinces during this period. It is possible that the 40 cases identified by the AFRIMS study are included in the 326 cases reported by the OLMIS dashboard. After simulating locations for these cases, these data were used to train the machine learning model for malaria niche estimation. Lastly, the road network data were obtained from Open Street Map (OSM) for Ubon Ratchathani and Sisaket, and used these data to estimate travel routes from home villages to estimated work locations of the survey participants.

Eight different remotely-sensed environmental data layers were used for malaria ecological niche estimation including total precipitation, landcover, population, elevation, slope, and average, minimum and maximum temperatures for both provinces. Precipitation estimates were obtained from WorldClim and included the monthly total amount of precipitation at a 21 km resolution, which was then averaged to create an annual precipitation layer for 2018 [28]. Landcover data are categorical and sourced from the Mekong Landcover Viewer [29]. From the original data, there are 20 different land cover classifications, however these classes were consolidated into nine classes by generalizing multiple forest classes to a general classification of forested areas. Shuttle Radar Topography Mission (SRTM) 2014 elevation data was used in this research, including for calculating slope using ArcGIS Pro 2.6.2 [30]. Temperature variables were annual averages for 2020 and were obtained from NASA’s Moderate Resolution Imaging Spectroradiometer (MODIS) [31]. Lastly, population density data were sourced from Oak Ridge National Laboratory's 2019 Landscan resource [32].

## Methodology

Using the malaria case data and the environmental data, the malaria parasite distribution was determined using an ecological niche model for the study area. The relationship between the ecological niche, and work locations and occupation types were analysed using a leave-one-out validation process to determine which variables have the strongest relationship with areas with a higher probability of malaria occurrence. To implement this process, a number of models were created. Each variable was excluded in turn, and a model created with the remaining variables. Then a model was created using each variable in isolation. In addition, a model was created using all variables, as before. The area under the ROC curve (AUC) values from all the results were compared to determine the relative strength of each variable.

As the cases provided through the OLMIS for Thailand were geolocated only to the district level, a random simulation workflow was conducted using R to generate estimates of home village locations for these malaria-positive individuals (Fig. 2).

The random simulation process was executed based on the following rules:

- Points were randomly generated within the district boundary;
- The number of points generated corresponded to the number of cases within each district;
- Locations with low residential population numbers based on Landscan 2019 population estimates were excluded, including the forested escarpment area along the Cambodia border;
- Uniform random sampling was used as the population distribution for the area is widely spread, with only a few urban areas with higher population density such as the City of Ubon Ratchathani, the former capital of the province, with a population of 79,000 (Department of Provincial Administration, 2014). However, the risk of contracting malaria in urban areas is likely to be less than in rural areas as health care providers tend to cluster in urban areas, and the lack of habitats for the vector and a lack of travel to rural areas in urban areas. Public health measures are more likely to be in place and to have sufficient funding to be maintained in urban areas [33, 34].

The generated points were used as occurrence data in the ecological niche model. One thousand different sets of random points by district were simulated in order to generate the highest level of randomness and to avoid bias in the results [35, 36]. All environmental variables were pre-processed using R to be the same projection, extent, and spatial resolution.

The MaxEnt modelling tool was run over 1000 iterations for each set of randomly generated home village locations and the eight pre-processed environmental layers in R. Three different training–testing splits were analysed to test the sensitivity of the models and evaluate the quality of the results including 70-30, 80-20 and 100% of the data as training data. The area under the curve (AUC) values for the 1000 iterations were averaged to determine the training–testing split that was used for the analyses.

A random sampling approach was used to estimate 100 work locations for rubber plantation workers and farmers. Farmers were assumed to have worked in both “Rice paddy” and “Cropland” regions while rubber plantation workers were assumed to work in “Forest” regions (Fig. 2). This assumption was tested using the demographic data collected by AFRIMS. The work locations were then generated using a uniform random sampling package from R package ‘sf’ applying these restrictions for the working regions for farmers and rubber plantation workers, respectively.

Logistic regression was applied to estimate the association between the estimated probability of malaria occurrence and occupation types. This study focused only on two occupations, farmers and rubber plantation workers, classified as categorical variables when building the model. These two occupation groups were tested because they comprised 65% of the collected data and represent different land cover classes (forested areas, croplands, and rice paddies), allowing us to analyse one group that works in forested areas and one that does not. A one-way Analysis of Variance (ANOVA) test was applied to determine the difference in malaria risk between different occupations. In this study, the probability of malaria was measured being present at a location as determined from the ecological niche model. This probability served as the dependent variable while occupation type was the independent variable. The ANOVA test made it possible to understand if there were significant differences in the probability of malaria at the work locations for either rubber plantation workers or farmers. The ecological niche model values were extracted at the estimated work locations obtained based on the Landcover data and analysed for the corresponding occupation type.

To further analyse the relationship between occupation-related factors and malaria occurrence, a road network and buffer analysis was conducted to determine the risk of exposure that could be associated with the travel routes to work. The shortest paths from home villages to the estimated work locations for the cases identified by AFRIMS were calculated using ArcGIS Pro 2.6.2 and the OSM street network. Using a 1 km buffer around work locations and a 500-m buffer that extended from the work location along the shortest path routes, these areas were also analysed with respect to their overlap with the niche areas.

## Results

Base on the AFRIMS survey data, the primary occupation types of those surveyed included farmers (35%), rubber plantation workers (30%), service workers (12.5%), forestry officers (10%) and monks (7.5%). When comparing the results of the three different training and testing split model runs for estimating ecological niche, there was little deviation in the average AUC values used to assess predictive accuracy, and the contributions of environmental variables. The average AUC differed less than 0.015 between the model runs, and the top contributing environmental variables were consistently elevation, average and maximum temperature, and landcover (Table 1). As the 70-30 training testing split results returned the highest accuracy of 79%, this split was used to further analyse occupation and travel in the context of ecological niche.

**Table 1:** Percent contributions of environmental variables used to train the ecological niche model

Environmental Variable	70-30 (%)	80-20 (%)	100 (%)
Elevation	49.61	49.82	49.57
Landcover	8.88	8.75	8.58
Population	0.99	0.99	0.97
Precipitation	3.6	3.57	3.23
Slope	3.45	3.35	3.27
Average temperature	16.76	16.45	17.36
Maximum temperature	10.84	11	11.02
Minimum temperature	5.85	6.01	6

**Fig. 3:**
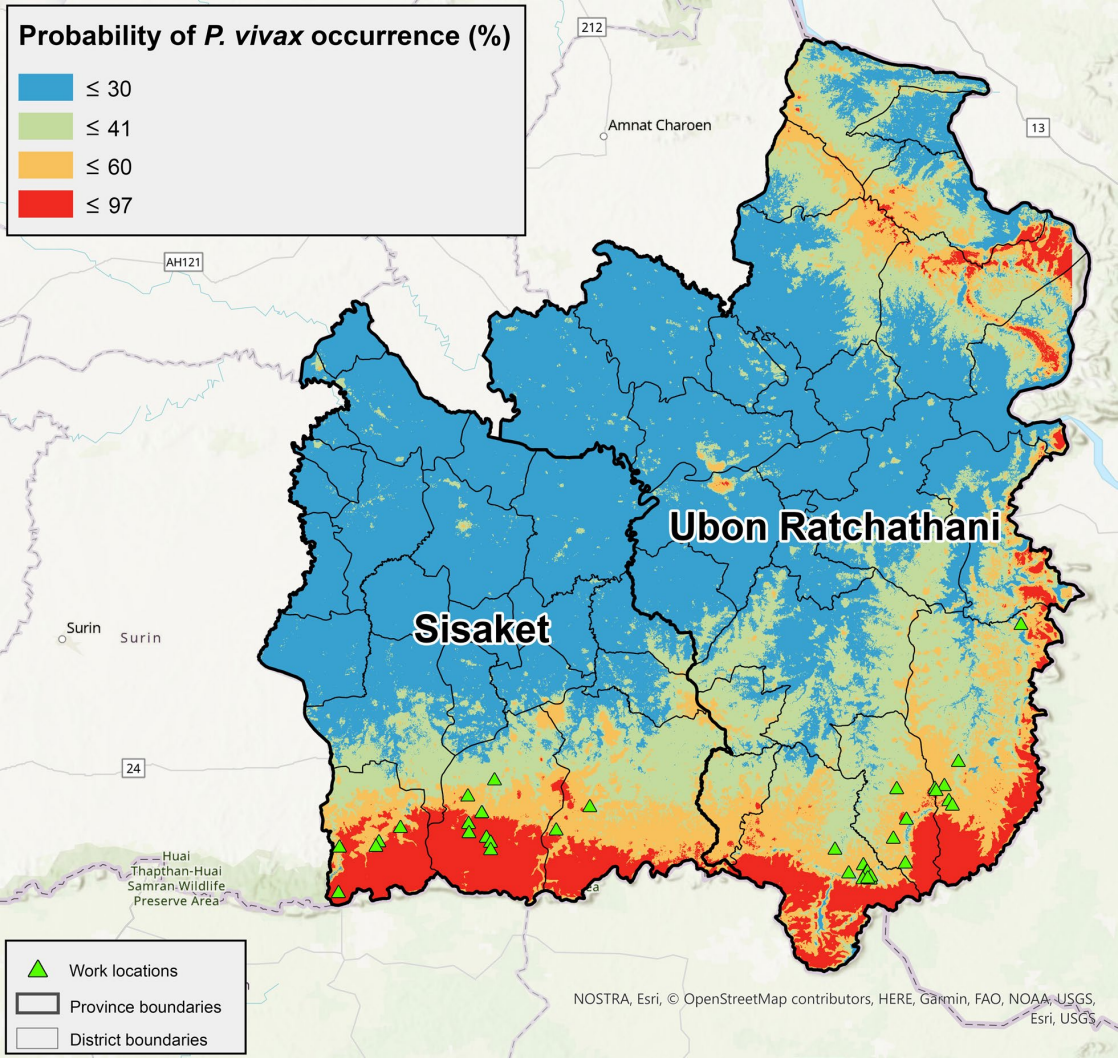
Estimated ecological niche and work locations based on the GPS coordinates from AFRIMS data

The estimated ecological niche results showed that the highest probability locations of malaria occurrence were located along the Thailand–Cambodia border, particularly in the forested escarpment area (Fig. 3). The highest probability locations were also detected for northern Ubon Ratchathani province near the Thailand-Lao border. Using a natural breaks classification, the model results were classified into four classes reflecting the probability of malaria occurrence and assigned work locations from AFRIMS survey data into a class based on the ecological niche model value at these locations. The results showed that the majority of the study sample population fell into class 3 (79%), where there was a 41–60% chance of malaria occurrence, with 95% of work locations falling in class 3 or 4 (Fig. 3). None of the work locations were found in a class 1 area, i.e., areas with the lowest probability of malaria occurrence.

To understand the potential malaria risk for rubber plantation workers and farmers, the simulated work locations for the two occupation groups were analysed using the landcover data and the study sample data was used to validate the simulation points. The results showed that 72% of simulated work locations for both farmers and rubber plantation workers were consistent with the reported work locations from the survey.

**Fig. 4:**
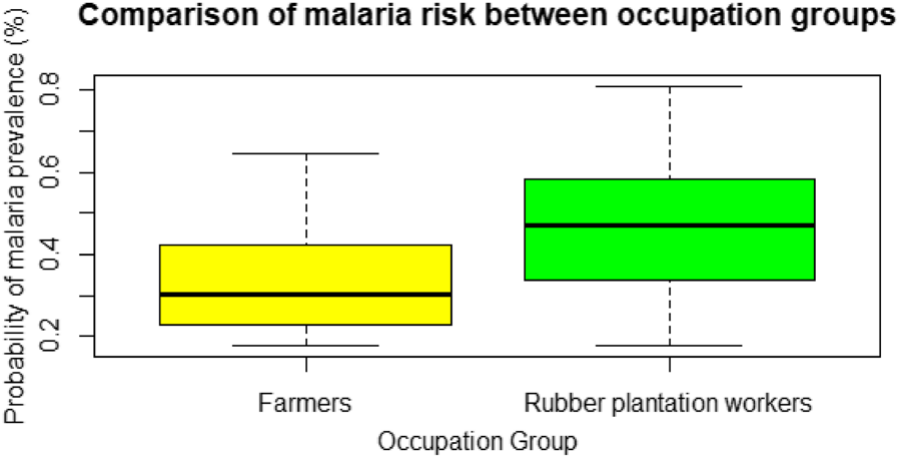
Comparison of average MaxEnt values by occupation type

A logistic regression analysis was applied to occupation type and predicted MaxEnt values. The outputs showed the coefficient for predicted MaxEnt value was positive with a coefficient of 9.03 and a p-value of 9.3e−10. The one-way ANOVA test returned a statistically significant difference between the average MaxEnt predicted values for the two occupation types of rubber plantation workers and farmers, returning a p-value of 1.73e−13. Based on the box-plot results, the average MaxEnt predicted values for rubber plantation workers were higher than those for farmers (Fig. 4). Rubber plantation workers were estimated to have significantly higher risk of exposure to malaria compared to farmers based on the predicted values from the machine learning analysis.

**Fig. 5:**
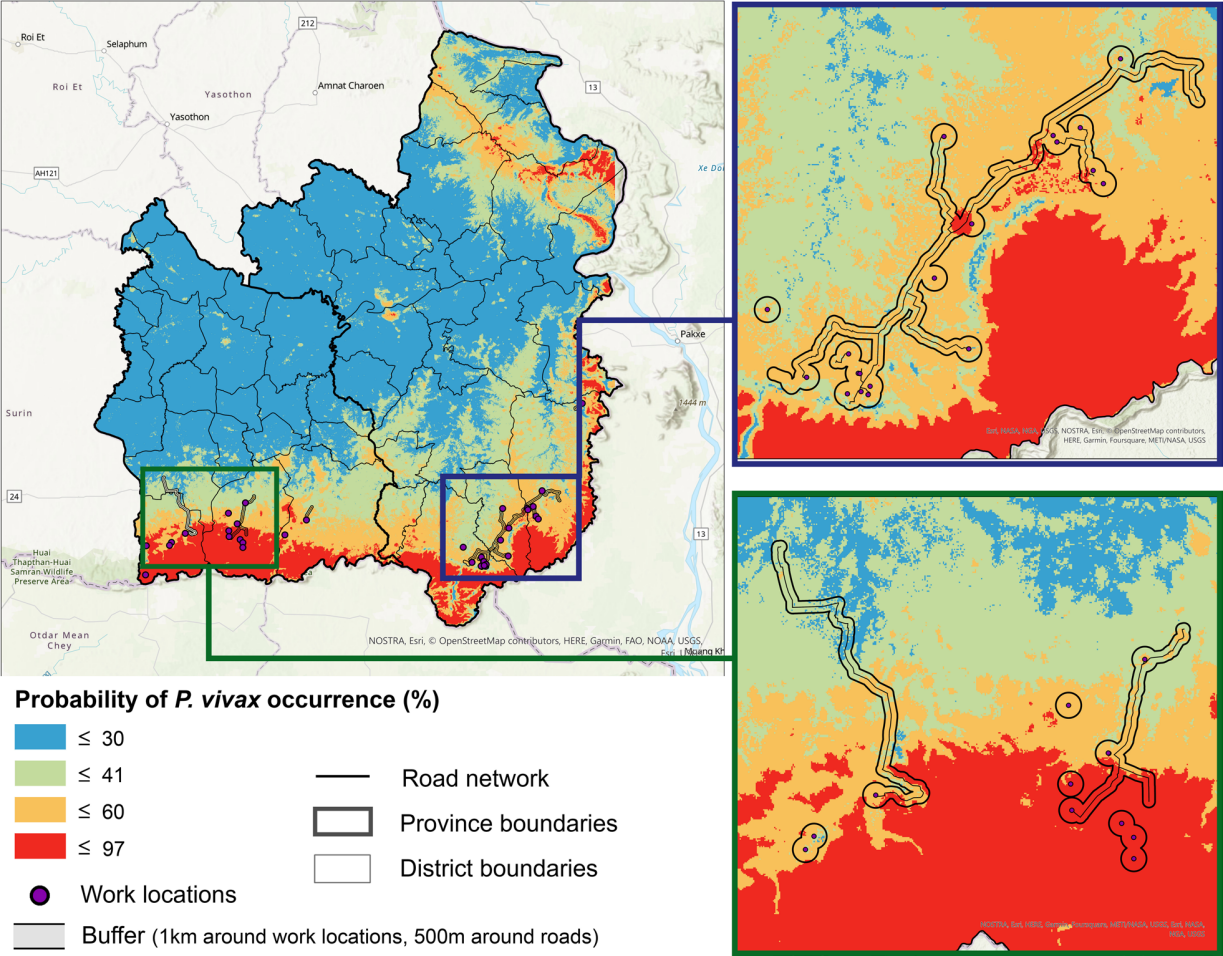
Analysing occupation-related travel routes and malaria occurrence probability using buffer analysis

Analysing the estimated travel routes between home village and work locations in the context of the niche extent showed that travel for the two occupation groups occurred in areas with higher probability of malaria occurrence (Fig. 5). The travel routes were found to go through areas corresponding to higher niche classes. This is important to understand because regular daily travel increases the chance of exposure to infection by travelling through higher probability areas of local malaria occurrence. When averaging model values within buffer areas, the results showed that rubber plantation workers were travelling in areas with higher probabilities of malaria occurrence (average ecological niche value of 0.515) slightly more than other occupations (average ecological niche value within buffers for all occupations of 0.503).

## Discussion

Computing the ecological niche showed higher probabilities of *P. vivax* occurrence was along the Thailand–Cambodia border. Additional high probability locations were detected in northern Ubon Ratchathani province near the Thailand-Lao border. The species distribution was modelled primarily for *P. vivax* malaria as there was a higher probability of *P. vivax* occurrence reflected by both the MOPH and AFRIMS data. This region of the world is also commonly referred to as having “border malaria”, i.e., a higher prevalence of malaria in Southeast Asia exists along international borders [2, 37, 38] and the niche analysis also returned this pattern of species distribution. Similar to the findings in this research, a study focused on the Buriram and Surin provinces of Thailand determined through multiple linear regression analysis that “the high-risk areas of malaria cases were on the Thai-Cambodian border” and that malaria morbidity rates were strongly associated with forested areas [39]. Further, in an overview of the epidemiological patterns of malaria in the Greater Mekong Subregion from 1998 to 2007, researchers found that malaria was most prevalent in forested and forest-fringe areas, primarily along international borders [40]. The niche model results also reflected these findings, as it similarly predicted higher probabilities of *P. vivax* occurrence in a similar ecological area. The red and orange regions of the border areas which are representing regions with high risk of malaria of the mapped niche shown in Figs. 4, 5, are also consistent with the active malaria transmission foci map for 2020 for Ubon Ratchathani and Sisaket as produced by the Sudathip et al. [41].

The ANOVA test results showed significant differences between forest and non-forest-based occupations, indicating a significant relationship between occupation type and working in an area of higher malaria occurrence. Further analysis showed that compared to farmers, rubber plantation workers were working in areas with a higher risk of malaria occurrence. This higher risk may be due to the fact that the working hours and outdoor practices of rubber plantation workers often overlap with peak mosquito biting times [42, 43].

Previous studies have found a relationship between occupation type and malaria risk. For example, out of over 4000 malaria cases reviewed in seven provinces of southern Thailand (Chumphon, Nakhon Si Thammarat, Krabi, Phangnga, Phuket, Ranong and Surat Thani provinces), nearly 62% malaria cases occurred in rubber plantation workers [44]. One study, based on qualitative ethnographic research of the Oddar Meanchey Province of Cambodia, found that those who worked in forests experienced multiple episodes of malaria more frequently than other occupations. Local residents understood the increased malaria risk, but for economic purposes, continued to pursue forest-based livelihoods. This geographic pattern is further supported by the analysis in this study, which estimated the highest probability of malaria occurrence in the forested areas along the Thailand–Cambodia border, and observed low but positive correlations between occupation types that worked in forests [45]. Researchers also found that forest-related occupations and economic drivers, such as logging of rosewood trees and other luxury timber, were the main reason for forest going which increase their exposure to malaria [46]. Similarly, another study conducted in Prachuap Khiri Khan Province of Thailand found that occupation as a rubber farmer/tapper was highly correlated with malaria affected households, and this correlation was statistically significant [47]. The same conclusion was drawn in this research that these same occupations worked in and travelled through areas with higher niche values and probabilities of malaria occurrence. The findings showed that likely daily travel routes take individuals from homes that may not be in a risk area to a work location that is in a risk area and that travel can be a possible driver for infection. Travel has been shown to play a key role in transmission [48] and, therefore, working in an area corresponding to a higher niche category, and travelling in and out, may increase the chance of transmission and infection. A recent study in Myanmar also found that forest workers have a higher malaria exposure compared with farmers as they were more likely to travel to regions with higher malaria prevalence for work [49].

One possible limitation of this research is that the analysis did not consider the presence of existing malaria control measures such as personal protection behaviours, or preventive measures for the simulation analysis. Likewise, the cases that might be due to staying outside at work locations were not considered. For the analysis of travel routes, although a 1 km buffer around work locations and a 500-m buffer along the travel routes were used to include more potential travel areas and to mitigate the uncertainty of the travel routes, these are estimates only and require more data to refine these values. For the AFRIMS survey data, the GPS coordinates were obtained using an approximate location near to the permanent residence in the village where they reside, and for work locations approximate geographic coordinates capturing a close location for where they worked were used. Future research could include ground truth data for the work locations and travel routes to validate the simulated routes used in this study. For example, GPS trajectory data can be used to provide more details about travel routes [50, 51]. For this study, district-level data was used when simulating the home locations for *P. vivax* cases from the OLMIS dataset. Future research could be undertaken at sub-district or village level data. More detailed and precise landcover data can also be used in the future to differentiate the working regions for rubber plantation workers and that of other forest workers. For this study, the possibility of recrudescence or relapse of *P. vivax* was not taken into account and for the AFRIMS survey data, it is not known if these were newly infected or recurrent cases. The data from the OLMIS dashboard also did not specify whether cases were first episodes or likely relapses. Distinguishing new infections from relapses is a continuing challenge in studies of *P. vivax*, and could have impacted the inferences.

## Conclusions

In this study, the probability of malaria occurrence across Ubon Ratchathani and Sisaket provinces of Thailand was examined. A maximum entropy model was developed that used the known locations of positive malaria cases sourced from OLMIS, to produce a mapped probability surface that represented the percent probability of malaria occurrence in these provinces. Other inputs to the model included environmental variables sourced from remotely sensed datasets including temperature, landcover, precipitation, and elevation, and population density, and these were used to train the machine learning model. The highest probability of malaria occurrence in Sisaket was found along the Thailand–Cambodia border. For Ubon Ratchathani, other than the Thailand–Cambodia border area, high malaria risk areas were also detected in the north of Ubon Ratchathani near the Thailand-Lao border.

An analysis of the relationship between occupation types and the model results returned a significant difference between the average probability of malaria occurrence for the work locations simulated using landcover data of different outdoor occupation groups. As part of this research, travel routes were generated and applied buffer analysis and then analysed these areas in the context of the probability of malaria occurrence to understand the relationship between daily work-related travel and the potential risk of exposure to malaria. The results showed that individuals working in and travelling to areas with higher probabilities of malaria occurrence were at higher risk, and that rubber plantation workers were travelling in areas with higher probabilities of malaria occurrence more frequently than other occupation types. Daily travel to work that takes individuals into areas with higher probabilities of malaria occurrence raises the risk of exposure relating to these travel behaviours.

## Data Availability

Landcover data is publicly available through http://servir-rlcms.appspot.com/static/html/home.html. Malaria cases data from MOPH is available through: http://malaria.ddc.moph.go.th/malariar10/index_newversion.php

## Abbreviations

ANOVA: Analysis of variance
AUC: Area under the curve
MOPH: The Ministry of Public Health for Thailand
OSM: Open street map
OLMIS: Online malaria information system
MaxEnt: Maximum entropy-based modelling
